# Language acquisition in a post-pandemic context: the impact of measures against COVID-19 on early language development

**DOI:** 10.3389/fpsyg.2023.1205294

**Published:** 2023-07-27

**Authors:** Sara Feijoo, Anna Amadó, Francesc Sidera, Eva Aguilar-Mediavilla, Elisabet Serrat

**Affiliations:** ^1^Department of Llengües i Literatures Modernes i Estudis Anglesos, Universitat de Barcelona, Barcelona, Spain; ^2^Department of Psicologia, Sociologia i Treball Social, Universitat de Lleida, Lleida, Spain; ^3^Department of Psicologia, Universitat de Girona, Girona, Spain; ^4^Department of Pedagogia Aplicada i Psicologia de l’Educació, Institut de Recerca i Innovació Educativa (IRIE), Universitat de les Illes Balears, Palma, Spain

**Keywords:** language acquisition, expressive vocabulary, parental questionnaires, pandemic, child development

## Abstract

Language acquisition is influenced by the quality and quantity of input that language learners receive. In particular, early language development has been said to rely on the acoustic speech stream, as well as on language-related visual information, such as the cues provided by the mouth of interlocutors. Furthermore, children’s expressive language skills are also influenced by the variability of interlocutors that provided the input. The COVID-19 pandemic has offered an unprecedented opportunity to explore the way these input factors affect language development. On the one hand, the pervasive use of masks diminishes the quality of speech, while it also reduces visual cues to language. On the other hand, lockdowns and restrictions regarding social gatherings have considerably limited the amount of interlocutor variability in children’s input. The present study aims at analyzing the effects of the pandemic measures against COVID-19 on early language development. To this end, 41 children born in 2019 and 2020 were compared with 41 children born before 2012 using the Catalan adaptation of the MacArthur-Bates Communicative Development Inventories (MB-CDIs). Results do not show significant differences in vocabulary between pre- and post-Covid children, although there is a tendency for children with lower vocabulary levels to be in the post-Covid group. Furthermore, a relationship was found between interlocutor variability and participants’ vocabulary, indicating that those participants with fewer opportunities for socio-communicative diversity showed lower expressive vocabulary scores. These results reinforce other recent findings regarding input factors and their impact on early language learning.

## 1. Introduction

It is a well-known fact that linguistic input is multimodal in nature, and that language learners make use of multimodal cues. While sound is the most obvious cue to speech comprehension in oral languages, since the McGurk effect was first described (McGurk and MacDonald, 1976), decades of research have shown that visual cues have an important impact in speech comprehension, and that infants use such cues from a very early age (Hollich et al., 2005; Bahrick and Lickliter, 2012; Kawase et al., 2014; Astor et al., 2021; Çetinçelik et al., 2021). For instance, young infants pay more attention to new vowel contrasts when these are presented in an audiovisual modality rather than when they are presented in an audio-only or a visual-only modality (Ter Schure et al., 2016). Also, young children show a preference for the speaker’s mouth rather than other areas of the face (Tenenbaum et al., 2015; Tsang et al., 2018).

Furthermore, research also shows that access to input variability seems fundamental for young learners, in order for them to acquire the patterns and rules of the language they are exposed to (Perry et al., 2010; Rowe, 2012; Jones and Rowland, 2017; Serrat et al., 2021; Kartushina et al., 2022). In particular, talker variability has been described to facilitate linguistic development (Richtsmeier et al., 2009; Rost and McMurray, 2009; Rojas et al., 2016). Richtsmeier et al. (2009) showed that exposure to nonwords spoken by 10 different talkers resulted in faster and more accurate production among young children than exposure to the same nonwords spoken by a single talker. In a similar line, Rojas et al. (2016) found that preschoolers’ expressive language skills benefit from access to input by different interlocutors, particularly interactions with older siblings and peers. Rost and McMurray (2009) also found that infants exposed to multiple speakers showed higher word discrimination rates than infants in a single-speaker condition.

The COVID-19 pandemic context and, especially, the measures against the virus adopted worldwide might have had an impact on children’s language development. On the one hand, the generalised use of masks diminishes the quality of linguistic input, since masks distort the acoustic speech signal and, besides, they reduce visual cues to speech. On the other hand, restrictions regarding social gatherings as well as frequent lockdown episodes might have altered the variability of input to which children were exposed.

The data available so far is controversial (LoBue et al., 2023). On the one hand, some studies do suggest significant developmental differences between babies born during the pandemic and babies born before. For instance, Shuffrey et al. (2022) found that pandemic infants had significantly lower scores for gross motor, fine motor, and personal-social skills. In their study, none of the participant mothers or babies had been infected with the virus. Thus, the authors claim that the developmental differences found are not due to the virus itself, but to the social measures adopted against the virus, and the environment that was created as a result. Deoni (2022) also found that children born during the pandemic had significantly lower verbal, non-verbal, and cognitive performance compared to pre-pandemic children. Furthermore, Deoni’s study also showed that SES, birth weight and gestation duration were protective factors, since children with lower SES, lower weight and/or shorter gestation were more affected. In a similar line, Frota et al. (2022) found that post-pandemic children exhibited lower performance at word segmentation tasks than pre-pandemic children.

On the other hand, some other studies have found no differences between pre- and post-Covid children (Wermelinger et al., 2022; Sperber et al., 2023). For instance, Mitsven et al. (2022) found that post-Covid children’s language production was unaffected by mask use in the preschool classroom, and that children could benefit from the language they were exposed to despite their teachers’ mask. In a similar line, Singh et al. (2021) also found no differences in children’s ability to locate a target word referent when the target word was presented by a speaker wearing a mask, compared to a speaker without mask.

Given the controversy of the existing data, the present study aims at further exploring the effects that measures against COVID-19 might have had on children’s lexical development. While most data available so far comes from experimental studies (Singh et al., 2021; Deoni, 2022) or classroom settings (Mitsven et al., 2022), the present study uses parental questionnaires to assess vocabulary growth among young learners in the pandemic context. The use of such an instrument will grant access to children’s linguistic production in a number of different communicative situations, often only available to parents, especially during the pandemic restrictions. Furthermore, this method will easily allow comparisons between data collected during the pandemic and normative data collected before the pandemic with exactly the same instrument. In particular, the present analysis aims at answering the following research questions:

RQ1: Will children born in the pandemic context show lower expressive vocabulary scores than children born before the pandemic?

RQ2: Will measures against COVID-19 (i.e., mask use and restrictions in terms of social interaction) relate to children’s expressive vocabulary scores as measured by parental questionnaires?

## 2. Method

### 2.1. Participants

The study included 82 participants (38 girls) between 8 and 30 months (M age = 19.83 months; *SD* = 5.26) from Catalan-speaking families. Participants were divided into two groups: the pre-Covid group (41 participants born before 2012) and the post-Covid group (41 participants born between 2019 and 2020).

The pre-Covid group was created by selecting a sub-sample from the normative sample of the Catalan MB-CDI (Serrat et al., 2022). To create this control group, we randomly selected those children who matched the post-Covid children in the following variables: (a) age; (b) sex; (c) prematurity status; (d) linguistic context (i.e., degree of exposure to languages other than Catalan, see Serrat et al., 2021); (e) birth order; and (f) education of mothers (see Table 1 for sociodemographic variables).

**Table 1:** Sociodemographic variables for pre- and post-Covid groups.

Variables	Pre-Covid	Post-Covid	Differences between groups
	*M* (*SD*)	*M* (*SD*)	*U* Mann Whitney or *χ*^2^
*N*	41	41	
Age (months)	19.83 (5.26)	19.83 (5.26)	*U* = 840, *p* = 1.0
Gender			
Male	22	22	*χ*^2^ = 0.000, *p* = 1.00
Female	19	19	
Prematurity			
Yes	0	0	-
No	41	41	
Weigh at birth	3.32 (0.4)	3.27 (0.48)	*U* = 606, *p* = 0.676
Birth order			
1st	32	31	
2nd	9	9	*χ*^2^ = 1.016, *p* = 0.602
3rd	0	1	
Education of mothers^a^			
Primary	0	0	
Secondary	5	4	*χ*^2^ = 0.188, *p* = 0.655
University	34	37	
Bilingualism			
No	18	17	*χ*^2^ = 0.345, *p* = 0.842
Familiar bilingualism (only one Catalan-speaking parent)	6	8	
Other contacts	17	16	
Otitis^a^			
Yes	8	9	*χ*^2^ = 0.046, *p* = 0.829
No	32	32	
Previous language difficulties			
Yes	1	0	*χ*^2^ = 1.012, *p* = 0.314
No	40	41	

a Lost data.

The participants in each of these two groups (pre- and post-Covid) were divided into two subgroups according to their chronological age. Thus, their families were given a different version of the instrument to complete: families of children between 8 and 18 months answered the Catalan adaptation of the McArthur-Bates CDI inventory 1 (i.e., words and gestures, MB-CDI: WG), and families of children between 16 and 30 months answered the inventory 2 (i.e., words and sentences, MB-CDI: WS). In this way, a total of 32 participants (16 pre- and 16 post-Covid) completed the MB-CDI: WG (M age = 14.39 months; *SD* = 2.120) and 50 participants (25 pre- and 25 post-Covid) completed the MB-CDI: WS (M age = 23.2 months; *SD* = 3.452). Regarding the SES of the families, measured on the basis of maternal education, 6.1% of the sample completed secondary studies, 3.7% post-secondary studies, and 90.2% university studies.

### 2.2. Instruments

The data for this study was obtained using the MacArthur-Bates Communicative Development Inventories (MB-CDIs) adapted to Catalan (Serrat et al., 2022). Of all the sections included in this instrument, the vocabulary section was considered, since it is common to both questionnaires. Parents had to indicate their child’s ability to understand or say a series of words in the case of the MB-CDI: WG, or just the ability to say the words in the case of the MB-CDI: WS. The MB-CDI: WG lists 423 words which are grouped into 19 categories, while the MB-CDI: WS lists 678 words which are grouped into 22 categories.

For the gathering of the child’s personal and socio-demographic data, the last part of the questionnaire was used. For the specific purpose of the present study, in order to analyse the impact of measures against COVID-19 on vocabulary development, the following two questions were added:

- How would you define your child’s variety of sociocommunicative interaction in the last 3 months?
  (1) Very little (2) Little (3) Average (4) Quite a lot (5) A lot.
- How often has your child been in contact with interlocutors wearing a mask since the beginning of COVID?
  (1) Never (2) Hardly ever (3) Sometimes (4) Often (5) Always.

### 2.3. Procedure

In order to administer the questionnaires to the families of the post-Covid group, 3 early childhood educational centers in Catalonia (Spain) were contacted during June and July 2021. Several waves of lockdowns and restrictions of different types (e.g., mobility, social gatherings…) had occurred in this area since the beginning of the pandemic. All educational centers were closed from March to September of 2020. When they reopened, mask use was compulsory for teachers at all educational levels until April 2022. In addition, lockdown episodes occurred whenever positive cases emerged within a group and, consequently, all students were sent home for quarantine.

We contacted the directing teams of the educational centers through email. We described the objectives of the study and the characteristics of the target sample and asked them to give a document with information about the study and a consent form to the families. Parents who gave written consent received the questionnaires in written format and were asked to return them within a week. Most children from the post-Covid group attended educational centers (73.1%). The rest were personal and professional contacts of the authors. All questionnaires were filled by either the child’s mother or by both parents.

The total vocabulary scores for each child were used to calculate their percentile of expressive vocabulary according to the normative scores of the test, and this percentile of vocabulary was taken as a dependent variable. Data were analyzed with SPSS version 25. The nonparametric Mann–Whitney U test was used to compare independent groups, as dependent variables (Total expressive vocabulary and Percentile of expressive vocabulary) did not show a normal distribution (Shapiro–Wilk *W* = 0.796, *p* < 0.001 and *W* = 0.946, *p* = 0.002, respectively). A Chi-square approach was used to compare the number of participants classified as “high vocabulary level” (percentile equal to or over 75), “typical development” (percentile between 26 and 74), and “low vocabulary level” (percentile equal to or lower than 25). Finally, two linear regression analyses, one introducing the “diversity of communicative interaction” and the other one “face mask use” were performed over the dependent variable “total expressive vocabulary” in the sample of post-Covid children.

## 3. Results

Results show equal exposure to face masks between children studied with MB-CDI: WG and MB-CDI: WS (*U* = 779.5, *p* = 0.833), and between children born in 2019 and 2020 (*U* = 195, *p* = 0.803). However, older children received more diverse social interactions than younger children (*U_2019-2020_* = 118, *p* = 0.017; *U_CDI.WG-CDI.WS_* = 115, *p* = 0.023).

Although the means of expressive vocabulary were lower in the post-Covid group than in the pre-Covid group, results did not show significant differences between both groups, neither in the total expressive vocabulary (*U* = 796, *p* = 0.680), nor in the percentile of expressive vocabulary (*U* = 712.5, *p* = 0.234) (see Table 2).

**Table 2:** Mean, standard deviation and independent group comparison between the pre- and post-Covid groups.

Variables	Pre-Covid		Post-Covid		Differences between groups
	*M* (*SD*)	Range	*M* (*SD*)	Range	*U* Mann Whitney or *χ*^2^
Total expressive vocabulary	155.5 (182.7)	0–571	140.4 (174.1)	0–653	*U* = 796, *p* = 0.680
Percentile expressive vocabulary	51.8 (29.3)	5–99	43.8 (27.3)	5–95	*U* = 712.5, *p* = 0.234

Also, the distribution of children in three groups (i.e., low vocabulary level, typical development, and high vocabulary level) through standardized data (percentile) of their total expressive vocabulary did not show significant differences (see Table 3). Nevertheless, the distribution approximates significance when only two groups were considered (*χ*^2^ = 2.53, *p* = 0.099) showing more children with high vocabulary level in the pre-Covid group and more children with low vocabulary level in the post-Covid group.

**Table 3:** Distribution of children regarding expressive vocabulary performance in the pre- and post-Covid groups.

Variables	Pre-Covid	Post-Covid	Differences between groups	*n*	*n*	*χ* ^2^
Total participants	41	41	
Low vocabulary level (percentile <25)	11	15	*χ*^2^ = 2.721, *p* = 0.257
Typical vocabulary development (percentile 26–74)	18	20	
High vocabulary level (percentile >75)	12	6	

*n*, number of children.

In order to know which variables affected the total expressive vocabulary of the children in the post-Covid group, we performed two regression analyses, one introducing “diversity of communicative interaction” and the other introducing “face mask use” as independent variables over the dependent variable “total expressive vocabulary.”

As can be seen in Table 4, face mask use cannot explain differences in the total expressive vocabulary. Nevertheless, diversity of communicative interaction explains 16.6% of the variability of the total expressive vocabulary.

**Table 4:** Regression analyses for measures against COVID-19 over total expressive vocabulary.

	Total expressive vocabulary			
Predictor	*β*	*∆R ^2^*	*F* or *t*	*p*
Model 1		0.166	8.946	0.005
Diversity of communicative interaction	0.432		2.99	0.005
Model 2		−0.011	0.118	0.732
Face mask use	−0.038		−0.344	0.732

## 4. Discussion

The present study employed parental questionnaires to assess children’s vocabulary development during the pandemic context. The instrument provided detailed and accurate data of children’s vocabulary knowledge, which allowed for direct comparison with similar data from pre-pandemic children.

In terms of this comparison, as expressed in our first research question, the analysis found no differences between children in the pre-Covid group, and the post-Covid group as far as expressive vocabulary is concerned. Although there seems to be a tendency to a distribution of children with higher scores in the pre-Covid group and lower scores in the post-Covid group, these differences did not reach statistical significance. Thus, the results obtained from parents’ questionnaires in a domestic context are similar to the results found in a preschool classroom context by Mitsven et al. (2022), who also found that pandemic children benefit from teacher’s input as well as pre-pandemic children. Therefore, as suggested by LoBue et al. (2023), measures against COVID-19 might have had an impact on caregivers’ socioemocional behaviour, but the measures seem to have had little or no effect on infants’ development. Alternatively, it might be the case that post-pandemic children are not developing worse than pre-pandemic children, but simply differently, and that those differences are reflected in other areas of language development (Frota et al., 2022; Shuffrey et al., 2022). It should also be born in mind that additional factors such as SES have been described as protective factors in the pandemic (Deoni, 2022), since children with higher SES outperform children with lower SES. Given the fact that most of the children in our sample belong to a high SES, this might have weakened the differences between our post-pandemic group and our pre-pandemic group. Unfortunately, the limitations of the present study and the size of the present sample did not allow for proper comparisons of groups with different SES. Our sample size did not allow for within-group comparisons considering other factors either, nor did it provide widely generalizable data. However, these results might be an important contribution to the field, given the uniqueness of the circumstances in which these data were collected.

Regarding our second research question, the present analysis found that interlocutor variability was related to children’s expressive vocabulary, which indicates that those participants with more frequent lockdown episodes and less opportunities for socio-communicative diversity showed lower expressive vocabulary scores. As the literature suggests, exposure to vocabulary items spoken by different talkers results in faster and more accurate development than exposure to the same items spoken by a single talker (Richtsmeier et al., 2009). In the same line, Rojas et al. (2016) or Serrat et al. (2021) also found that preschoolers with access to input by different interlocutors show higher rates of expressive language skills, due to the wider range of topics, referents and vocabulary items that children are exposed to and, subsequently, acquire. However, an important limitation regarding the present study lies in the way sociocommunicative diversity was measured, given the fact that the question that was addressed to parents in the questionnaire might have been interpreted differently by different participants. Given the importance of sociocommunicative diversity and the relationship it seems to have with linguistic development, further research should explore this relationship with a more accurate and objective operationalization of the variable related to communicative diversity.

Regarding the use of mask, there seems to be no relationship between this measure against COVID-19 and children’s vocabulary development. Therefore, the present results are in line with those obtained by Singh et al. (2021), who also found no differences in terms of word-object identification between speech with mask or without mask. As some researchers have claimed (Pycha et al., 2022; Wermelinger et al., 2022), speakers might modify their language production in the presence of a physical barrier, namely a face mask, in order to make their speech more intelligible. In fact, previous findings have already shown that speakers tend to increase their speech quality (i.e., speech rate, pitch, length of words, etc.) while wearing a mask, in order to compensate for difficult communicative situations (Crimon et al., 2022). Additionally, non-verbal cues such as co-speech gestures, beats or iconic gestures have also been said to compensate for speech degradation in a number of contexts (Drijvers and Özyürek, 2017; Crimon et al., 2022). Therefore, it is possible that the use of alternative communication strategies might have neutralized the possible negative effects that masked speech might have produced otherwise.

## 5. Conclusion

The present study made use of parental questionnaires in order to assess expressive vocabulary development among children born within the COVID-19 pandemic context. As an instrument, the parental questionnaire provided valuable evidence of linguistic development from a sample in a context that was otherwise very difficult to obtain. At the same time, it allowed for direct comparisons with normative data obtained from children born before the pandemic. The main findings of such comparison revealed no significant differences between pre- and post-pandemic children in terms of expressive vocabulary. Nevertheless, further analyses within the post-pandemic group indicated that, despite mask-use had no effect on vocabulary development during the pandemic, restrictions on social gatherings did, given that lower interlocutor variability scores among post-pandemic children correlated with lower expressive vocabulary scores. Given the results obtained, future studies should further explore the relationship between interlocutor variability and early language development in order to confirm this finding.

## Data availability statement

The raw data supporting the conclusions of this article will be made available by the authors, without undue reservation.

## Ethics statement

Ethical review and approval was not required for the study on human participants in accordance with the local legislation and institutional requirements at the moment of the first data collection. Written informed consent to participate in this study was provided by the participants’ legal guardian/next of kin.

## Author contributions

SF: data collection, analysis and writing, and edition of the final paper. AA and FS: data collection, analysis, and writing of the final paper. EA-M and ES: data collection, analysis and writing of the final paper. All authors contributed to the article and approved the submitted version.

## Funding

This work was supported by grant 2021 SGR 00303 from the Department of Research and Universities of the Government of Catalonia.

## Conflict of interest

The authors declare that the research was conducted in the absence of any commercial or financial relationships that could be construed as a potential conflict of interest.

## Publisher’s note

All claims expressed in this article are solely those of the authors and do not necessarily represent those of their affiliated organizations, or those of the publisher, the editors and the reviewers. Any product that may be evaluated in this article, or claim that may be made by its manufacturer, is not guaranteed or endorsed by the publisher.
